# Assessment of Dietary Habits Using the Diet Quality Index—International in Cerebrovascular and Cardiovascular Disease Patients

**DOI:** 10.3390/nu13020542

**Published:** 2021-02-07

**Authors:** In Young Cho, Kyung Min Lee, Yoojin Lee, Chuel Min Paek, Ha Jin Kim, Ju Young Kim, Kiheon Lee, Jong Soo Han, Woo Kyung Bae

**Affiliations:** 1 Department of Family Medicine, Kangbuk Samsung Hospital, Sungkyunkwan University School of Medicine, Seoul 03181, Korea; inyoungs.cho@samsung.com; 2 Department of Family Medicine, Seoul National University Hospital, Seoul 03080, Korea; lkmlove88@naver.com (K.M.L.); l20121223gh@gmail.com (Y.L.); whitepc1222@naver.com (C.M.P.); hamayjin25@gmail.com (H.J.K.); 3 Department of Family Medicine, Seoul National University Bundang Hospital, Seongnam 13620, Korea; kkamburi@gmail.com (J.Y.K.); keyhoney@gmail.com (K.L.); 4 Health Promotion Center, Seoul National University Bundang Hospital, Seongnam 13620, Korea; hanjongsoomd@gmail.com

**Keywords:** cerebrovascular disorders, cardiovascular diseases, diet, nutrients, dietary habit

## Abstract

Improvement of dietary habits is recommended for the management of cerebrovascular and cardiovascular diseases (CCVD). This study aimed to evaluate the dietary habits of CCVD patients and compare them with the general population by using the Diet Quality Index—International (DQI-I). Data from the Korean National Health and Nutrition Examination Surveys (2013–2016) were used. Cardiovascular diseases included myocardial infarction, angina pectoris, and heart failure; and cerebrovascular diseases included stroke, cerebral infarction, and hemorrhage. In total, 12,683 subjects over 20 years old were included, comprising 718 CCVD patients and 11,965 non-CCVD subjects. Survey-weighted multiple linear regression analyses with adjustment for covariates were used to compare DQI-I scores. The mean total DQI-I scores for the CCVD and non-CCVD groups were 66.7 ± 9.2 and 67.8 ± 9.2, respectively. After adjusting for covariates, the CCVD group had DQI-I scores significantly lower than the non-CCVD group (coefficient −1.13, *p*-value = 0.011). In the analysis of each DQI-I component, the CCVD group had lower scores for variety (coefficient −0.54, *p*-value = 0.004) and adequacy (coefficient −0.86, *p*-value = 0.001). In this study, using nationally representative data, dietary habits of CCVD patients were shown to be lower in quality than non-CCVD subjects. Therefore, evaluation and education of adequate dietary habits are needed in the management of CCVD patients.

## 1. Introduction

A rapidly aging population and westernized lifestyle, including dietary habits, have led to increased cerebrovascular and cardiovascular disease (CCVD) mortality in South Korea. According to cause of death statistics released by Statistics Korea in 2017, cardiac diseases (including myocardial infarction, heart failure, and cardiac arrest) were the second leading cause of death after cancer, and cerebrovascular diseases (including stroke, infarction, and hemorrhage) were the third most common cause of death [1]. Hypertension, diabetes, and dyslipidemia are major risk factors for CCVD, and they are closely related to lifestyle behaviors, which if properly managed can help prevent up to 80% of premature deaths due to myocardial infarction or stroke [2]. CCVD patients are at high risk of recurrent cardiovascular or cerebrovascular events [3,4], and require secondary prevention. Unhealthy dietary habits, lack of exercise, smoking, and excessive alcohol drinking are known risk factors of CCVD, and interventions to improve these lifestyle behaviors are both feasible and necessary not only for primary prevention [5], but also for secondary prevention and management of CCVD [6,7].

Previous studies have demonstrated that improvement in dietary habits can have a positive effect on CCVD, and is helpful in their prevention and management [8,9,10,11,12]. Low-fat diets, low-carbohydrate diets, and the Mediterranean and dietary approaches to stop hypertension diets are some well-known examples of such diets [8,13,14,15,16]. Prospective studies have also shown that lifestyle changes, including dietary changes, may have a beneficial effect on CCVD patients [17,18]. However, there is insufficient information on the status of dietary habits of CCVD patients in South Korea.

Among the many methods available to evaluate dietary habits, the Diet Quality Index—International (DQI-I) allows for detailed evaluation of dietary habits, including factors closely related to chronic diseases and malnutrition, and for comparisons of diverse populations and countries [19]. A recent study in South Korea comparing dietary habits between cancer survivors and the general population confirmed that the DQI-I is a useful tool to reflect the dietary habits of both populations [20].

This study aimed to analyze dietary patterns of CCVD patients and compare them with non-CCVD subjects through the DQI-I by using nationally representative data, in order to evaluate the appropriateness of their dietary habits and demonstrate whether dietary interventions are needed in this population.

## 2. Materials and Methods

### 2.1. Study Participants

This study used data from the sixth and seventh Korean National Health and Nutrition Examination Surveys (KNHANES), comprising the years 2013 to 2016. The KNHANES is a study conducted annually by the Korean Ministry of Health and Welfare’s Centers for Disease Control and Prevention, providing cross-sectional data on the health status, health behaviors, and dietary habits of the general population. This study complied with the Helsinki Declaration as revised in 1983, and informed consent was waived by our Institutional Review Board (IRB) because it used data without personally identifiable information, provided by the KNHANES for public use (IRB No. X-1908-556-903).

Out of a total of 31,098 study participants, only those who reported that they had followed their diet as usual on the previous day were included, and those who reported that they had eaten in excess or less than their usual diet were excluded (*n* = 12,112). Subjects meeting the following criteria were also excluded: Younger than 20 years (*n* = 4173), unknown status of CCVD diagnosis (*n* = 1851), and missing data on the variables analyzed (*n* = 279). Subjects who responded that they were diagnosed with a cardiovascular or cerebrovascular disease were included in the CCVD group, and all the subjects responded that they had been diagnosed over a year ago. CCVD included both cardiovascular diseases (including myocardial infarction, angina pectoris, and heart failure) and cerebrovascular diseases (stroke, cerebral infarction, and hemorrhage). From a total of 12,683 subjects, 718 were assigned to the CCVD group, and 11,965 to the non-CCVD group (Supplementary Figure S1).

### 2.2. Measurements

Demographic characteristics including age, sex, educational level, monthly income, residential area, marriage status, health behaviors such as smoking and drinking, and diagnosis and treatment for diseases were collected through self-reported questionnaires. Body mass index (BMI) was calculated after measuring height and weight, and <18 kg/m^2^ was defined as underweight, 18–22.9 kg/m^2^ as normal, 23–24.9 kg/m^2^ as overweight, and ≥25.0 kg/m^2^ as obese, according to the World Health Organization criteria for defining obesity in Asians [21]. Previous studies have shown that all-cause mortality risk in Asians is equivalent to Caucasians at lower BMI (around 5 units), and risk increases starting at BMI ≥25.0 kg/m^2^ [22]. Marital status was defined as being unmarried, married with a spouse, or married without a spouse (due to divorce, separation, or death). Educational level was classified as elementary school or below, middle or high school graduate, and college graduate or above. Monthly income was divided into three groups: <1 million Won (November 2020, 1000 Korean Won = 0.90 USD), 1–3 million Won, and >3 million Won. Residential area was classified as urban or rural area. Smoking status was classified as current smoker, ex-smoker, or never smoker, and drinking patterns were classified as current drinkers, ex-drinkers or never drinkers. Chronic diseases included hypertension, diabetes, dyslipidemia, chronic renal disease, and osteoarthritis, and were evaluated as existing number of chronic diseases.

### 2.3. Evaluation of Dietary Habits

Information on dietary habits and food intake were collected through food frequency questionnaires (FFQ) and 24-h dietary recalls (24HR), which were administered once by trained dieticians through face-to-face interviews [23]. The FFQ in the KNHANES has acceptable reproducibility and modest validity for estimating usual dietary intake [24], and the 24HR was supervised by the dieticians. Using this information, the diet quality of the study subjects was evaluated through the DQI-I. The DQI-I includes the following four components: Variety (20 points), adequacy (40 points), moderation (30 points), and overall balance (10 points), and the sum of these components adds to a total of 100 points, with higher scores meaning better diet quality [19]. The evaluation of the percentage of each nutrient intake, which reflects the adequacy of diet, was performed using the Reference Intakes for Koreans published by the Korean Nutrition Society in 2015 [25]. Empty calorie foods were high calorie foods made up of mostly carbohydrate and fats, and low in essential nutrients such as vitamins or amino acids, such as ice cream and cookies [19,20].

### 2.4. Statistical Analysis

Survey-weighted univariate logistic and linear regressions were performed to compare the general characteristics between the CCVD and non-CCVD groups, and linear regressions were performed to compare the DQI-I scores between the 2 groups.

Survey-weighted univariate linear regressions were performed to examine the associations between the DQI-I and covariates, and variables that showed statistical correlation with the DQI-I were integrated into the multiple linear regression models comparing the DQI-I scores between the CCVD and the non-CCVD groups. Model 1 was unadjusted, and model 2 was adjusted for age and BMI as continuous variables, and for sex, smoking status, alcohol consumption, and number of comorbidities as categorical variables. Model 3 was adjusted for the covariates in model 2, and also for socioeconomic factors including educational level, monthly income, residential area, and marriage status, which were shown to be associated with the DQI-I through univariate analysis. Statistical significance for each DQI-I component was corrected with Bonferroni’s penalization method; *p*-values were determined as 0.05 divided by the number of components. Because there are four main DQI-I components, statistical significance was set at *p*-value <0.0125 (equal to 0.05 divided by 4) for the four DQI-I components. For the 17 subcategories within the DQI-I components, statistical significance was set at *p*-value <0.003 (equal to 0.05 divided by 17). Graphical data were expressed with figures using R statistical software version 3.5 (R Foundation for Statistical Computing, Vienna, Austria) and Rex version 3.0.3 (Rexsoft Co. Ltd., Seoul, South Korea), and all statistical analyses were performed using Stata version 15.0 (Stata Corp., College Station, TX, USA), setting statistical significance at *p*-value <0.05 unless otherwise specified.

## 3. Results

### 3.1. Study Population Characteristics

The mean age was 68.8 ± 8.9 years in the CCVD group, and 52.3 ± 16.4 years in the non-CCVD group. The mean BMI was 24.6 ± 3.4 kg/m^2^ in the CCVD group, and 23.7 ± 3.5 kg/m^2^ in the non-CCVD group. The non-CCVD group was more highly educated and had a higher monthly income than the CCVD group, and a higher percentage lived in urban areas. The non-CCVD group was also more likely to be current smoker or drinker. However, the CCVD group was more likely to have more than one chronic disease. General characteristics of the CCVD and non-CCVD groups can be found in Table 1.

### 3.2. Univariate Analysis of Diet Quality Using the DQI-I

**Figure 1:**
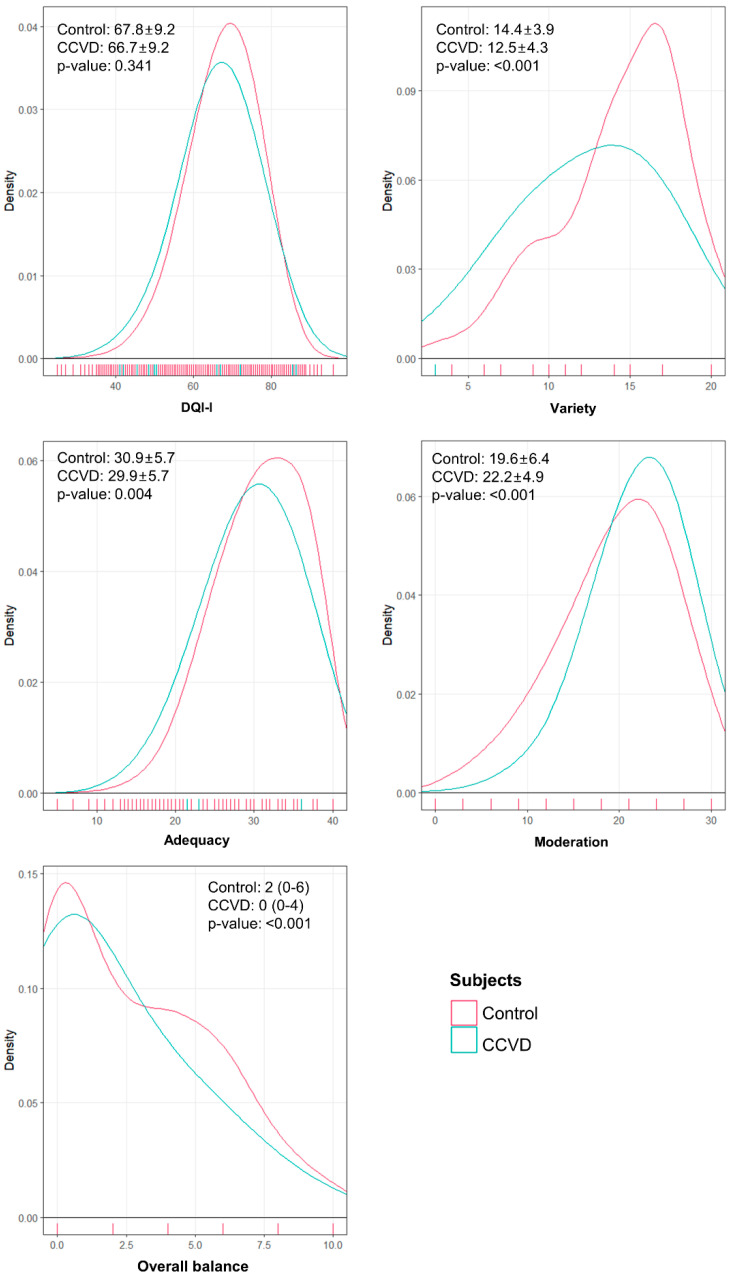
DQI-I component score densities. DQI-I, Diet Quality Index—International; CCVD, cerebrovascular and cardiovascular diseases. Total DQI-I, variety, adequacy, and moderation scores are presented as means and standard deviations. Overall balance scores are presented as medians with interquartile ranges. *p*-values were obtained by survey-weighted linear regressions.

The mean total DQI-I score for the CCVD group was 66.7 ± 9.2, and 67.8 ± 9.2 for the non-CCVD group (*p*-value = 0.341). Variety and adequacy scores were lower in the CCVD group than in the non-CCVD group (variety 12.5 ± 4.3 vs. 14.4 ± 3.9, *p*-value <0.001; adequacy 29.9 ± 5.7 vs 30.9 ± 5.7, *p*-value = 0.004). Overall balance scores were also lower in the CCVD group, with median and interquartile range (IQR) scores of 0 (0–4) in the CCVD group and 2 (0–6) in the non-CCVD group (*p*-value <0.001). Moderation scores were higher in the CCVD group (22.2 ± 4.9 vs 19.6 ± 6.4, *p*-value <0.001). The distribution and scores of total DQI-I and its components can be found in Figure 1.

### 3.3. Adjusted Analysis of DQI-I Scores

Comparisons of total DQI-I scores between the CCVD and non-CCVD groups after adjustment for covariates are shown in Table 2. The CCVD group’s DQI-I scores were significantly lower than the non-CCVD group in both model 2 and model 3 after adjusting for covariates (model 2: β = −1.96, *p*-value <0.001; model 3: β = −1.13, *p*-value = 0.011).

### 3.4. Adjusted Analysis of DQI-I Components

Components of the DQI-I score were also compared after adjusting for covariates including age, sex, BMI, marriage status, education level, monthly household income, residential area, smoking, alcohol consumption, and number of chronic diseases, as shown in Table 3. The CCVD group was found to have significantly lower scores than the non-CCVD group for variety (β = −0.54, *p*-value = 0.004), adequacy (β = −0.86, *p*-value = 0.001), fiber (β = −0.22, *p*-value <0.001), and iron (β = −0.14, *p*-value = 0.001).

## 4. Discussion

The present study compared dietary habits between CCVD patients and non-CCVD subjects by comparing DQI-I scores, with the aim to assess the diet quality of CCVD patients and search for areas in need of improvement. The CCVD group had lower diet quality than the non-CCVD group, with lower total DQI-I scores and lower scores for variety and adequacy.

Cerebrovascular and cardiovascular diseases are a major cause of death in South Korea, accounting for approximately one quarter of all deaths, and associated medical costs and socioeconomic burdens are rapidly increasing [1,26]. One of the most effective strategies for both primary and secondary prevention of CCVD is management of risk factors such as hypertension, diabetes, dyslipidemia, and abdominal obesity, and major guidelines around the world also recommend adherence to healthy dietary habits [5,27]. CCVD patients have a high risk of subsequent CCVD events, and since evidence suggests that combinations of dietary changes may lead to improved outcomes [17], they should adhere to healthy diets for secondary prevention [6]. Recent guidelines from the American College of Cardiology and American Heart Association [5], the European Society of Cardiology [7], and the World Health Organization [27] all recommend following a varied diet including vegetables, fruits and seaweeds, and plenty of milk and milk processed foods for prevention and management of cardiovascular diseases. Saturated fatty acids and trans fatty acids should be replaced with unsaturated fatty acids such as fish and nuts, and salt intake should be restricted to a decent amount, along with adequate intake of micronutrients and avoidance of soft drinks [5,7,27,28].

In this study, although the CCVD group was found to have lower scores for the total DQI-I and its components compared to the non-CCVD group, the difference itself was marginal, as shown in Figure 1. Among the DQI-I analyses shown in Table 2, the lowest total DQI-I score for the CCVD group compared to the non-CCVD group can be found in model 2, which was adjusted for age. Several studies have pointed out that older age is associated with higher diet quality, probably because older people are more likely to follow the traditional Korean diet [29], characterized by a higher intake of vegetables and lower in fats than the Western diet, which in turn can lead to improved diet quality [30,31]. Meanwhile, age is also a risk factor for CCVD [32]. The mean age of the CCVD group was about 15 years older than the non-CCVD group (68.8 ± 8.9 vs. 52.3 ± 16.4 years), which may have affected the DQI-I scores before adjustment. Therefore, it is necessary to control for age effects by appropriately matching for age and other covariates in studies that compare CCVD patients with other non-CCVD subjects. In our study, CCVD subjects had lower total DQI-I scores even after adjustment for age and other covariates.

Although not statistically significant, regression coefficients for moderation subcategory scores, in particular for total fat, cholesterol, and salt intake, showed positive associations for the CCVD group, meaning that CCVD patients may be following diets low in salt and cholesterol. This may be due to the emphasis in guidelines for chronic diseases on lowering fat, cholesterol, and salt intake [33,34,35,36]. Further efforts may be needed in clinical practice to educate patients on dietary habits, such as adding variety to their diets by increasing not only vegetable and fruit intake, but also consuming adequate amounts of dietary fiber and micronutrients. However, the positive correlations in cholesterol and salt intake may have been caused by recall or social desirability bias, because results were based on self-reported dietary questionnaires.

This study had several strengths. First of all, it used nationally representative data of the Korean population to compare dietary habits between CCVD patients and non-CCVD subjects. Second, it used a diet quality index that can evaluate dietary habits through specific measurements of not only dietary nutrients, but also other components such as variety and balance, and could be used for comparisons between different populations.

However, this study also had several limitations. First of all, it was a cross-sectional study, and therefore was limited in its ability to explain causal relationships between CCVD diagnosis and dietary habits. Further large-scale prospective studies are needed in order to assess the association between CCVD diagnosis and dietary habits. Second, the cross-sectional design of our study led to differences in age, sex, and lifestyle behaviors between the CCVD and non-CCVD groups. Although we adjusted for these confounding factors in our analyses, further prospective studies matching for age and other confounding factors may minimize the confounding effect of these covariates. Third, the FFQ and 24HR were conducted only once to assess dietary habits. These questionnaires require cognitive skills; respondents have to recall what they ate and report it appropriately [37]. Information on dietary intake may need to be collected repeatedly across several non-consecutive days, to reliably collect information on usual food intake and dietary habits. Recall or social desirability bias may also interfere with accurate collection of data, including information on lifestyle behaviors, which were collected through self-administered questionnaires. Finally, our study was conducted using the KNHANES, and therefore may not be generalizable to populations of other cultures or ethnicities.

## 5. Conclusions

CCVD patients are at high risk of recurrent cardiovascular or cerebrovascular events, and should adhere to healthy lifestyle behaviors, including healthy dietary habits, for secondary prevention. Our study results using a nationally representative database suggest that the dietary quality of CCVD patients is lower than that of non-CCVD subjects. Therefore, physicians may be encouraged to evaluate the dietary quality of CCVD patients during hospital visits, and educate them to improve their adherence to adequate diets.

## Figures and Tables

**Table 1:** General characteristics of study subjects.

	Total (*n* = 12,683)	CCVD (*n* = 718)	Non-CCVD (*n* = 11,965)	*p*-Value ^1^
**Age (y)**	53.3 ± 16.5	68.8 ± 8.9	52.3 ± 16.4	<0.001
**19–29**	1172 (9.2)	1 (0.1)	1171 (9.8)	<0.001
**30–39**	1862 (14.7)	2 (0.3)	1860 (15.6)	
**40–49**	2196 (17.3)	17 (2.4)	2179 (18.2)	
**50–64**	3618 (28.5)	181 (25.2)	3437 (28.7)	
**≥65**	3835 (30.2)	517 (72.0)	3318 (27.7)	
**Sex**				
**Male**	5325 (42.0)	388 (54.0)	4937 (41.2)	<0.001
**Female**	7358 (58.0)	330 (46.0)	7028 (58.7)	
**BMI (kg/m^2^)**	23.8 ± 3.5	24.6 ± 3.4	23.7 ± 3.5	<0.001
**Underweight (<18)**	545 (4.3)	20 (2.8)	525 (4.4)	<0.001
**Normal weight (18–22.9)**	4979 (39.3)	210 (29.3)	4769 (39.9)	
**Overweight (23–24.9)**	3005 (23.7)	180 (25.1)	2825 (23.6)	
**Obese (≥25)**	4154 (32.8)	308 (43.0)	3846 (32.1)	
**Spouse**				
**With spouse**	9171 (72.3)	506 (70.5)	8665 (72.4)	<0.001
**Without spouse**	1947 (15.4)	202 (28.1)	1745 (14.6)	
**Unmarried**	1565 (12.3)	10 (1.4)	1555 (13.0)	
**Education**				
**≥College**	3749 (29.6)	56 (7.8)	3693 (30.9)	<0.001
**Middle/high school**	5521 (43.5)	265 (36.9)	5256 (44.0)	
**≤Elementary school**	3413 (26.9)	397 (55.3)	3016 (25.2)	
**Monthly income**				
**>3000**	5853 (46.2)	162 (22.6)	5691 (47.6)	<0.001
**1000–3000**	4237 (33.4)	242 (33.7)	3995 (33.4)	
**<1000**	2593 (20.4)	314 (43.7)	2279 (19.1)	
**Residential area**				
**Urban**	10,057 (79.3)	524 (73.0)	9533 (79.7)	0.002
**Rural**	2626 (20.7)	194 (27.0)	2432 (20.3)	
**Smoking**				
**Current smoker**	2139 (16.9)	113 (15.7)	2026 (16.9)	0.01
**Ex-smoker**	2685 (21.2)	250 (34.8)	2435 (20.4)	
**Never smoker**	7859 (62.0)	355 (49.4)	7504 (62.7)	
**Alcohol**				
**Current drinker**	8603 (67.8)	382 (53.2)	8221 (68.7)	<0.001
**Ex-drinker**	8890 (18.1)	193 (26.9)	2097 (17.5)	
**Never drinker**	1790 (14.1)	143 (19.9)	1647 (13.8)	
**Chronic disease**				
**0**	7508 (59.2)	125 (17.4)	7383 (61.7)	<0.001
**1**	2732 (21.5)	221 (30.8)	2511 (21.0)	
**2**	1624 (12.8)	200 (27.9)	1424 (11.9)	
**≥3**	819 (6.5)	172 (24.0)	647 (5.4)	

Values are presented as means and standard deviations, or numbers and percentages. ^1^ *p*-values were calculated by survey-weighted univariate logistic regressions for categorical variables, and survey-weighted univariate linear regressions for continuous variables. CCVD, cerebrovascular and cardiovascular diseases; BMI, body mass index.

**Table 2:** Multivariate analysis for DQI-I associated with CCVD.

Model	Regression Coefficient	95% CI	*p*-Value
**Model 1**	−0.43	−1.32 ~ 0.46	0.341
**Model 2**	−1.96	−2.89 ~ −1.02	<0.001
**Model 3**	−1.13	−2.00 ~ −0.26	0.011

Survey-weighted multiple linear regressions were performed. Model 1 was unadjusted. Model 2 was adjusted for age, sex, body mass index, smoking status, alcohol drinking, and number of comorbid chronic diseases. Model 3 was adjusted for variables in model 2 and marriage status, education, monthly income, and residential area. DQI-I, Diet Quality Index—International; CCVD, cerebrovascular and cardiovascular diseases; CI, confidence interval.

**Table 3:** Multivariate analysis for DQI-I components associated with CCVD.

DQI-I Components	Regression Coefficient	95% CI	*p*-Value
**Variety**	−0.54	−0.90 ~ −0.17	0.004
Overall food group variety	−0.38	−0.63 ~ −0.13	0.003
Within-group variety	−0.16	−0.29 ~ −0.17	0.028
**Adequacy**	−0.86	−1.38 ~ −0.34	0.001
Vegetable	−0.04	−0.10 ~ 0.02	0.206
Fruit	−0.12	−0.31 ~ 0.07	0.201
Grain	−0.01	−0.06 ~ 0.04	0.769
Fiber	−0.22	−0.34 ~ −0.97	<0.001
Protein	−0.03	−0.10 ~ 0.04	0.433
Iron	−0.14	−0.22 ~ −0.06	0.001
Calcium	−0.10	−0.22 ~ 0.03	0.124
Vitamin C	−0.21	−0.37 ~ −0.05	0.012
**Moderation**	0.32	−0.12 ~ 0.76	0.159
Total fat	0.04	−0.08 ~ 0.17	0.509
Saturated fat	−0.02	−0.14 ~ 0.09	0.697
Cholesterol	0.24	0.08 ~ 0.40	0.004
Sodium	0.19	−0.04 ~ 0.42	0.100
Empty calorie foods	−0.13	−0.34 ~ 0.09	0.238
**Overall balance**	−0.05	−0.31 ~ 0.21	0.704
Macronutrient ratio	−0.02	−0.23 ~ 0.18	0.824
Fatty acid ratio	−0.03	−0.18 ~ 0.12	0.721

Survey-weighted multiple linear regressions were performed adjusting for age, sex, body mass index, marriage status, education, monthly income, residential area, smoking status, alcohol drinking, and number of comorbid chronic diseases. DQI-I, Diet Quality Index—International; CCVD, cerebrovascular and cardiovascular diseases; CI, confidence interval.

## Data Availability

Publicly available datasets were analyzed in this study. The data can be found here: https://knhanes.cdc.go.kr/knhanes/main.do (accessed on 20 November 2018).

## Supplementary Materials

The following are available online at https://www.mdpi.com/2072-6643/13/2/542/s1, Figure S1: Flowchart of study participants’ inclusion and exclusion criteria, Table S1: Diet Quality Index—International (DQI-I) component scores in cerebrovascular and cardiovascular disease (CCVD) patients and non-CCVD subjects.

## Abbreviations

CCVD	Cerebrovascular and cardiovascular disease
DQI-I	Diet Quality Index—International
KNHANES	Korean National Health and Nutrition Examination Survey
IRB	Institutional Review Board
BMI	Body mass index
FFQ	Food frequency questionnaire
24HR	24-h dietary recall
IQR	Interquartile range

## References

[1] Korean Statistical Information Service Annual Report on the Cause of Death Statistics. http://kosis.kr.

[2] World Health Organization (2005). Preventing chronic diseases: A vital investment. WHO Global Report.

[3] Kerr A.J., Broad J., Wells S., Riddell T., Jackson R. (2009). Should the first priority in cardiovascular risk management be those with prior cardiovascular disease?. Heart.

[4] Feng W., Hendry R.M., Adams R.J. (2010). Risk of recurrent stroke, myocardial infarction, or death in hospitalized stroke patients. Neurology.

[5] Arnett D.K., Blumenthal R.S., Albert M.A., Buroker A.B., Goldberger Z.D., Hahn E.J., Himmelfarb C.D., Khera A., Lloyd-Jones D., McEvoy J.W. (2019). 2019 ACC/AHA Guideline on the Primary Prevention of Cardiovascular Disease: A Report of the American College of Cardiology/American Heart Association Task Force on Clinical Practice Guidelines. Circulation.

[6] Virani S.S., Smith S.C., Stone N.J., Grundy S.M. (2020). Secondary Prevention for Atherosclerotic Cardiovascular Disease: Comparing Recent US and European Guidelines on Dyslipidemia. Circulation.

[7] Piepoli M.F., Hoes A.W., Agewall S., Albus C., Brotons C., Catapano A.L., Cooney M.T., Corra U., Cosyns B., Authors/Task Force Members (2016). 2016 European Guidelines on cardiovascular disease prevention in clinical practice: The Sixth Joint Task Force of the European Society of Cardiology and Other Societies on Cardiovascular Disease Prevention in Clinical Practice (constituted by representatives of 10 societies and by invited experts): Developed with the special contribution of the European Association for Cardiovascular Prevention & Rehabilitation (EACPR). Eur. J. Prev. Cardiol..

[8] Eilat-Adar S., Sinai T., Yosefy C., Henkin Y. (2013). Nutritional recommendations for cardiovascular disease prevention. Nutrients.

[9] Hu F.B., Willett W.C. (2002). Optimal diets for prevention of coronary heart disease. JAMA.

[10] Ornish D., Scherwitz L.W., Billings J.H., Brown S.E., Gould K.L., Merritt T.A., Sparler S., Armstrong W.T., Ports T.A., Kirkeeide R.L. (1998). Intensive lifestyle changes for reversal of coronary heart disease. JAMA.

[11] Van Horn L., McCoin M., Kris-Etherton P.M., Burke F., Carson J.A., Champagne C.M., Karmally W., Sikand G. (2008). The evidence for dietary prevention and treatment of cardiovascular disease. J. Am. Diet. Assoc..

[12] Parikh P., McDaniel M.C., Ashen M.D., Miller J.I., Sorrentino M., Chan V., Blumenthal R.S., Sperling L.S. (2005). Diets and cardiovascular disease: An evidence-based assessment. J. Am. Coll. Cardiol..

[13] Tsai A.G., Wadden T.A. (2006). The evolution of very-low-calorie diets: An update and meta-analysis. Obesity (Silver Spring).

[14] Katcher H.I., Hill A.M., Lanford J.L., Yoo J.S., Kris-Etherton P.M. (2009). Lifestyle approaches and dietary strategies to lower LDL-cholesterol and triglycerides and raise HDL-cholesterol. Endocrinol. Metab. Clin. N. Am..

[15] Mozaffarian D., Katan M.B., Ascherio A., Stampfer M.J., Willett W.C. (2006). Trans fatty acids and cardiovascular disease. N. Engl. J. Med..

[16] Dansinger M.L., Gleason J.A., Griffith J.L., Selker H.P., Schaefer E.J. (2005). Comparison of the Atkins, Ornish, Weight Watchers, and Zone diets for weight loss and heart disease risk reduction: A randomized trial. JAMA.

[17] Iestra J.A., Kromhout D., van der Schouw Y.T., Grobbee D.E., Boshuizen H.C., van Staveren W.A. (2005). Effect size estimates of lifestyle and dietary changes on all-cause mortality in coronary artery disease patients: A systematic review. Circulation.

[18] Chow C.K., Jolly S., Rao-Melacini P., Fox K.A., Anand S.S., Yusuf S. (2010). Association of diet, exercise, and smoking modification with risk of early cardiovascular events after acute coronary syndromes. Circulation.

[19] Kim S., Haines P.S., Siega-Riz A.M., Popkin B.M. (2003). The Diet Quality Index-International (DQI-I) provides an effective tool for cross-national comparison of diet quality as illustrated by China and the United States. J. Nutr..

[20] Lee Y., Koo H.Y., Cho I.Y., Jo M., Kim K.C., Eum Y.H., Kim J.Y., Lee K., Lee K.H., Jung S.Y. (2019). Dietary Patterns Assessed by the Diet Quality Index-International Among Cancer Survivors Compared with Healthy Control Subjects: Using the Korea National Health and Nutrition Examination Surveys 2013–2015. KJFP.

[21] World Health Organization, Regional Office for the Western Pacific (2000). The Asia-Pacific Perspective: Redefining Obesity and Its Treatment.

[22] Wen C.P., David Cheng T.Y., Tsai S.P., Chan H.T., Hsu H.L., Hsu C.C., Eriksen M.P. (2009). Are Asians at greater mortality risks for being overweight than Caucasians? Redefining obesity for Asians. Public Health Nutr..

[23] Kweon S., Kim Y., Jang M.-j., Kim Y., Kim K., Choi S., Chun C., Khang Y.-H., Oh K. (2014). Data Resource Profile: The Korea National Health and Nutrition Examination Survey (KNHANES). Int. J. Epidemiol..

[24] Kim D.W., Song S., Lee J.E., Oh K., Shim J., Kweon S., Paik H.Y., Joung H. (2015). Reproducibility and validity of an FFQ developed for the Korea National Health and Nutrition Examination Survey (KNHANES). Public Health Nutr..

[25] The Korean Nutrition Society Dietary Reference Intakes for Koreans. http://www.kns.or.kr/FileRoom/FileRoom_view.asp?idx=79&BoardID=Kdr.

[26] Ministry of Health and Welfare Chronic Disease Status and Issues. http://www.cdc.go.kr/contents.es?mid=a20303020300.

[27] World Health Organization (2007). Prevention of Cardiovascular Disease: Guidelines for Assessment and Management of Total Cardiovascular Risk.

[28] Drewnowski A. (2005). Concept of a nutritious food: Toward a nutrient density score. Am. J. Clin. Nutr..

[29] Kang M., Joung H., Lim J.H., Lee Y.-S., Song Y.J. (2011). Secular Trend in Dietary Patterns in a Korean Adult Population, Using the 1998, 2001, and 2005 Korean National Health and Nutrition Examination Survey. Korean J. Nutr..

[30] Kim S.H., Oh S.Y. (1996). Cultural and nutritional aspects of traditional Korean diet. World Rev. Nutr. Diet..

[31] Lee M.J., Popkin B.M., Kim S. (2002). The unique aspects of the nutrition transition in South Korea: The retention of healthful elements in their traditional diet. Public Health Nutr.

[32] Anderson K.M., Odell P.M., Wilson P.W.F., Kannel W.B. (1991). Cardiovascular disease risk profiles. Am. Heart J..

[33] Korean Academy of Medical Sciences Evidence-Based Guideline for Hypertension in Primary Care. https://www.cdc.go.kr/board/board.es?mid=a20503050000&bid=0021&tag=&act=view&list_no=127643.

[34] Korean Academy of Medical Sciences Evidence-Based Guideline for Type 2 Diabetes in Primary Care. https://www.cdc.go.kr/board/board.es?mid=a20503050000&bid=0021.

[35] Rhee E.J., Kim H.C., Kim J.H., Lee E.Y., Kim B.J., Kim E.M., Song Y., Lim J.H., Kim H.J., Choi S. (2019). 2018 Guidelines for the management of dyslipidemia. Korean J. Intern. Med..

[36] Seo M.H., Lee W.Y., Kim S.S., Kang J.H., Kang J.H., Kim K.K., Kim B.Y., Kim Y.H., Kim W.J., Kim E.M. (2019). 2018 Korean Society for the Study of Obesity Guideline for the Management of Obesity in Korea. J. Obes. Metab. Syndr..

[37] Schatzkin A., Kipnis V., Carroll R.J., Midthune D., Subar A.F., Bingham S., Schoeller D.A., Troiano R.P., Freedman L.S. (2003). A comparison of a food frequency questionnaire with a 24-hour recall for use in an epidemiological cohort study: Results from the biomarker-based Observing Protein and Energy Nutrition (OPEN) study. Int. J. Epidemiol..

