# Supplementary material for: Assessment of Dietary Habits Using the Diet Quality Index—International in Cerebrovascular and Cardiovascular Disease Patients
Source: Nutrients. 2021 Feb 7;13(2):542. doi: 10.3390/nu13020542 (PMC7914702; doi:10.3390/nu13020542)
Supplement: Supplementary file 1 [file nutrients-13-00542-s001.pdf]

nutrients

MDPI

Supplementary Materials

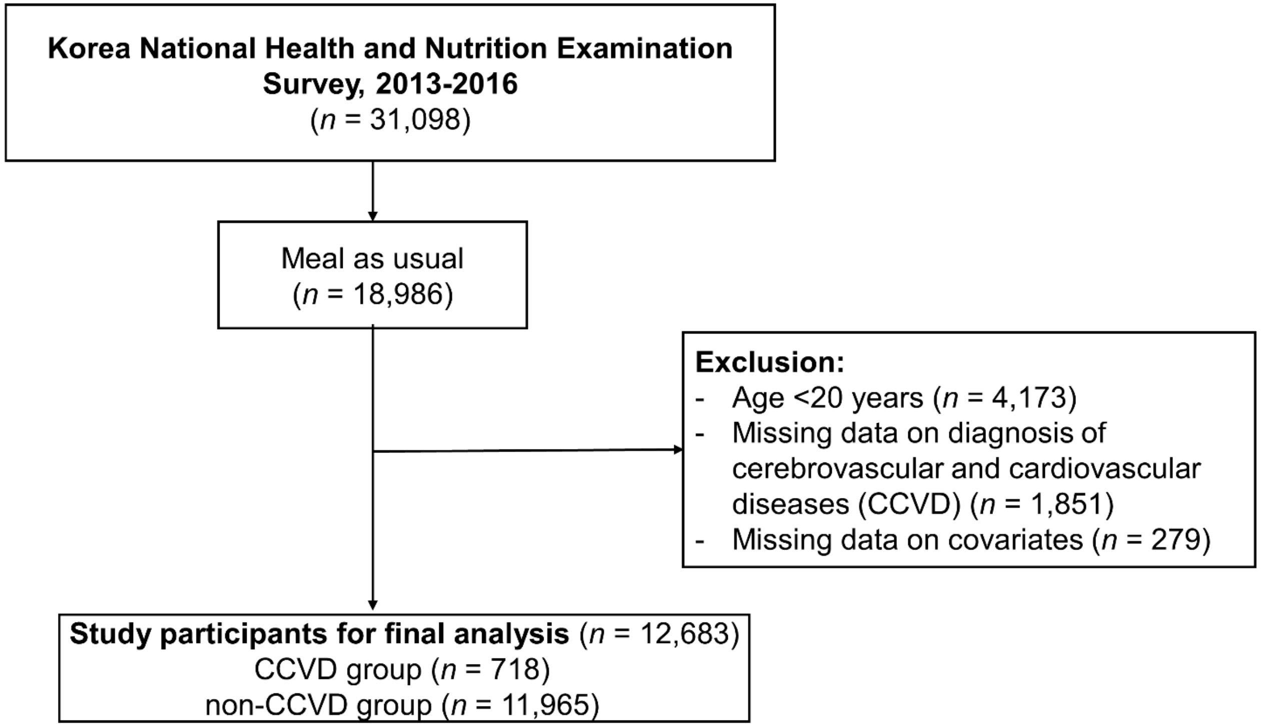

Figure S1. Flowchart of study participants' inclusion and exclusion criteria.

Table S1. Diet Quality Index-International (DQI-I) component scores in cerebrovascular and cardiovascular disease (CCVD) patients and non-CCVD subjects.

DQI-I components (highest points)	Total (*n* = 12,683)	CCVD (*n* = 718)	Non-CCVD (*n* = 11,965)	*p*-value [1]
**DQI-I overall score (100)**	67.71 ± 9.16	66.72 ± 9.23	67.77 ± 9.15	0.003
**Variety (20)**	14.31 ± 3.90	12.53 ± 4.26	14.41 ± 3.85	<0.001
overall food group variety (15)	10.17 ± 2.84	8.85 ± 2.97	10.25 ± 2.82	<0.001
within-group variety (5)	4.13 ± 1.37	3.67 ± 1.55	4.16 ± 1.35	<0.001
**Adequacy (40)**	30.80 ± 5.65	29.89 ± 5.65	30.86 ± 5.65	<0.001
vegetable (5)	4.84 ± 0.70	4.84 ± 0.72	4.84 ± 0.70	0.824
fruit (5)	2.73 ± 2.09	2.45 ± 2.10	2.75 ± 2.09	<0.001
grain (5)	4.9 ± 0.50	4.90 ± 0.50	4.90 ± 0.50	0.925
fiber (5)	3.91 ± 1.34	3.73 ± 1.41	3.92 ± 1.33	<0.001
protein (5)	4.70 ± 0.72	4.57 ± 0.83	4.71 ± 0.71	<0.001
iron (5)	4.46 ± 1.03	4.60 ± 0.89	4.46 ± 1.04	<0.001
calcium (5)	2.37 ± 1.36	2.09 ± 1.37	2.39 ± 1.36	<0.001
vitamin C (5)	2.87 ± 1.75	2.72 ± 1.74	2.88 ± 1.75	0.018
**Moderation (30)**	19.77 ± 6.33	22.17 ± 4.88	19.63 ± 6.38	<0.001
total fat (6)	4.72 ± 1.94	5.40 ± 1.35	4.68 ± 1.97	<0.001
saturated fat (6)	5.15 ± 1.73	5.62 ± 1.20	5.13 ± 1.75	<0.001
cholesterol (6)	4.76 ± 2.25	5.39 ± 1.67	4.72 ± 2.27	<0.001
sodium (6)	2.66 ± 2.62	3.13 ± 2.66	2.63 ± 2.62	<0.001
empty calorie foods (6)	2.48 ± 2.35	2.62 ± 2.32	2.47 ± 2.35	0.108
**Overall balance (10)**	2 (0–6)	0 (0–4)	2 (0–6)	<0.001
macronutrient ratio (6)	0 (0–4)	0 (0–2)	0 (0–4)	<0.001
fatty acid ratio (4)	0 (0–2)	0 (0–2)	0 (0–2)	0.030

Data are presented as means and standard deviations, or as medians and interquartile ranges. [1] Student's t-tests were performed for comparisons between CCVD patients and non-CCVD subjects; except for overall balance, where Wilcoxon rank-sum tests were performed.
